# Visual cues elicit differential aggression towards female and female mimics in the corkwing wrasse

**DOI:** 10.1093/beheco/arag022

**Published:** 2026-02-25

**Authors:** Benjamin A Ellis, Tonje K Sørdalen, Mark Briffa, Anne Berit Skiftesvik, Alexander D M Wilson, Kim A T Halvorsen

**Affiliations:** School of Biological and Marine Sciences, Portland Square, University of Plymouth, Devon PL4 8AA, United Kingdom; Institute of Marine Research, Flødevigen, Nye Flødevigveien 20, N-4817, Norway; Institute of Marine Research, Flødevigen, Nye Flødevigveien 20, N-4817, Norway; Centre for Coastal Research, Department of Natural Sciences, University of Agder, Kristiansand N-4604, Norway; School of Biological and Marine Sciences, Portland Square, University of Plymouth, Devon PL4 8AA, United Kingdom; Institute of Marine Research, Austevoll Research Station, Sauganeset 16, Storebø N-5292, Norway; School of Biological and Marine Sciences, Portland Square, University of Plymouth, Devon PL4 8AA, United Kingdom; Institute of Marine Research, Flødevigen, Nye Flødevigveien 20, N-4817, Norway

**Keywords:** alternative reproductive tactics (ARTs), female mimicry, nest defense, parental care, recognition, sexual dimorphism, video monitoring, visual cues

## Abstract

Alternative reproductive tactics (ARTs) are consistent intrasexual differences in behavior that maximize reproductive success through different means. In corkwing wrasse (*Symphodus melops*), smaller males mimic female visual morphology (hereafter sneakers) to steal fertilizations from nest-building parental males (hereafter nesting males). We investigated whether this visual mimicry effectively deceived 47 nesting males across 4 sites by quantifying 2 agonistic responses (attack latency and attack frequency) to models depicting unfamiliar females, sneakers and rival nesting males. Contrary to expectations, nesting males exhibited faster agonistic responses towards female models, with lower attack latencies compared with both male types. Additionally, the relationship between model type and attack frequency varied between sites, with reduced attack frequencies at sites subject to increased levels of anthropogenic disturbance. These results provide evidence that nesting males can visually discriminate between females and sneakers even in the absence of behavioral cues, suggesting that visual mimicry alone may be insufficient for successful deception. This highlights the need to investigate alternative sensory modalities, such as behavioral mimicry, to explain the successful reproduction of sneaker males and the maintenance of ARTs in this species.

## Teaser

In corkwing wrasse (*Symphodus melops*) sneaker males mimic females to steal fertilizations from parental nesting males, avoiding the energetic costs associated with extensive parental care. However, we found that nesting males can visually recognize sneaker males despite their mimicry of female visual traits. These findings highlight the remarkable visual recognition ability of nesting males, while suggesting that sneaker males use additional strategies and/or forms of mimicry to successfully reproduce.

## Introduction

Alternative reproductive tactics (ARTs) can be defined as consistent within-sex variations in reproductive behavior employed by individuals to enhance reproductive success under competitive conditions (Taborsky 2008; Kustra and Alonzo 2020). These tactics are most commonly found when conspecific competitors can exploit the investment of other individuals to improve their own fitness (Taborsky 2008) and are widespread across a myriad of invertebrate and vertebrate taxa (Oliveira et al. 2008; Garner and Neff 2020). Alternative tactics can be fixed, exhibited simultaneously, or arise sequentially throughout an individual's life (Taborsky 2008), resulting in the expression of a range of often correlated—behavioral, physiological and morphological polymorphisms. Mechanistically, these polymorphisms can have an underlying genetic basis or arise and be maintained through condition dependence in natural populations (Taborsky 2008).

Widespread among teleost fishes, ARTs predominantly occur as territorial and sneaker tactics in males (Taborsky 1997, 2001; Mank et al. 2006; Mank and Avise 2006; Oliveira et al. 2008; Zarini et al. 2023). In these systems, territorial males will invest in diverse strategies to monopolize access to critical reproductive resources such as higher quality territories, females, or fertilizations (Blumer 1979, 1982; McKaye et al. 1990; Rossiter 1995; Martin and Taborsky 1997; Rossiter and Yamagishi 1997; Taborsky 2001; Sefc 2011; Svensson and Kvarnemo 2023). They may also invest in the growth and maintenance of secondary sexual characteristics to attract mates and deter competitors (Andersson 1994; Taborsky 1994; Kodric-Brown 1998; Houde 2001; Maan and Seehausen 2011; Butts et al. 2012; Ota and Kohda 2015; Ciccotto and Mendelson 2016a, 2016b; Ota 2019; Wheeler et al. 2020). In some cases, territorial individuals, also referred to as nesting males, will build energetically costly structures (eg nests) to attract mates and facilitate brood care (Blumer 1979; Clutton-Brock 1991; Taborsky 2008; Stiver and Alonzo 2011; Svensson and Kvarnemo 2023), while also provisioning or protecting offspring during development (Blumer 1979, 1982; Taborsky 2008; Stiver and Alonzo 2011; Goldberg et al. 2020). Conversely, sneaker males invest in behavioral, morphological and physiological traits that enable them to steal fertilizations through various strategies involving surprise or deception (Dominey 1980; Gonçalves et al. 1996; Taborsky 2008; Gonçalves et al. 2024) and/or increased sperm investment (Taborsky 1998; Neff et al. 2003; Stoltz and Neff 2006a, 2006b; Taborsky 2008; Kustra and Alonzo 2020; Dougherty et al. 2022).

One such trait is female mimicry which has evolved repeatedly in many species exhibiting sexual dimorphism between territorial males and females (Andersson 1994; Taborsky 1994; Gross 1996; Taborsky 1997, 2001, 2008). Through mimicking female characteristics such as size, shape, coloration and/or behavior, sneakers can deceive territorial males allowing them to steal fertilizations during gamete release (Dominey 1980; Taborsky 1994; Gonçalves et al. 2024). However, the degree of visual mimicry varies greatly between species; some mimic relatively few female characteristics, such as imitating a specific pattern or coloration while maintaining other male or sneaker specific features (Dominey 1980; Kano et al. 2006; Rios-Cardenas et al. 2010). In other species, female visual characteristics are mimicked so effectively that they can only be identified through key behaviors, gametes or dissection (Dipper and Pullin 1979; Dominey 1980; Gross 1982; Uglem et al. 2000; Gonçalves et al. 2005; Stoltz and Neff 2006a). Territorial males incur a reproductive cost by losing fertilizations to sneaker males, with this cost being especially high in species with parental care. As a result, territorial males are under selection for traits enabling sneaker recognition, while selection acts on sneaker males to avoid detection. This arms race results in a high diversity of tactics and countermeasures between sneaker and territorial males, yet the sensory mechanisms that underpin recognition and deception in these complex behavioral interactions are seldom studied, confounding the understanding of these systems, a problem that is magnified by the brevity of these interactions, and the difficulty human observers face reliably differentiating sneaker males from females in certain species.

The corkwing wrasse (*Symphodus melops*) is a polymorphic species, that relies on visual information for foraging and courtship, exhibiting 3 morphs (nesting males, females and sneaker males), which are fixed from an early life stage (Potts 1974; Potts 1985; Hughes and Blight 2000; Loew et al. 2016). The relative proportions of these morphs can vary considerably at both large and small spatial scales, with sneaker males outnumbering nesting males in some populations (Uglem et al. 2000; Halvorsen et al. 2016). Likewise, life span is also spatially variable ranging from 4 to 9 years depending on the region (Darwall et al. 1992; Halvorsen et al. 2016). Territorial nesting males are larger than females and during the mating season from May to July, will invest in vivid blue and orange nuptial coloration that is unique to the individual (Halvorsen et al. 2016; Olsen et al. 2023). During this period nesting males will compete for territory before constructing elaborate domed nests out of various algal species with a small depressed entrance on one side, which are then aggressively defended (Potts 1974, 1985; Karaszkiewicz 2020). Simultaneously, females invest in bright yellow coloration and develop prominent blue genital papilla, while sneaker males mimic these features to an extremely high degree of accuracy. Nesting males will then engage in courtship with females within the nest, culminating in the simultaneous release of gametes (Potts 1974). After fertilization nesting males will engage in parental care behaviors, oxygenating the eggs via pectoral fin fanning, maintaining the nest structure and investing in vigilance behaviors to defend the eggs from predators including conspecific females (Potts 1974, 1985; Karaszkiewicz 2020; Ellis 2023). Fertilized eggs will hatch after 9 to 14 days, following which the nesting male will start a new nesting cycle, building up to 3 nests per season (Potts 1985). In order to steal fertilizations from nesting males during gamete release sneaker males employ 2 tactics, either rapidly entering the nest at the moment of gamete release, known as *bomb-sneaking* or *streaking* (Potts 1974; Taborsky 2008; Karaszkiewicz 2020; Ellis 2023); or through mimicking the courtship behavior of a female to gain access to the nest during or soon after courtship, before releasing sperm to fertilize the newly released eggs, referred to as *sneaking* (Potts 1974; Taborsky 2008; Karaszkiewicz 2020; Ellis 2023).

The high degree of visual mimicry observed in sneaker males suggests that it plays a key role in successful sneaking behavior. However, even though sneaker males and females cannot be distinguished by human experts, it is possible that the nesting males are able to do so (Bradbury and Vehrencamp 1998; Loew et al. 2016; Ciccotto and Mendelson 2016b; Caves et al. 2019; Cheney et al. 2019). Here we sought to test the effectiveness of the mimicry of female visual characteristics of sneaker males by comparing the agonistic behaviors of nesting males when presented with models depicting unknown female and sneaker male morphs. A previous study (Ellis 2023) demonstrated that nesting males of *S. melops* will respond aggressively when presented with a model of another intruder nesting male near their nest. This approach of quantifying agonistic responses to model intruders can be further adapted to investigate the ability of nesting males to discriminate between sneaker males and females on the basis of visual cues alone. If nesting males are unable to discriminate between sneaker males and females, they should show equal levels of (low) agonistic responses to models depicting females and sneakers relative to models of nesting males. Alternatively, if they can discriminate between sneakers and females, both the sneaker male models and nesting male model should elicit greater agonistic responses compared with female models.

## Materials and methods

### Data collection

#### Collection site

**Figure 1:**
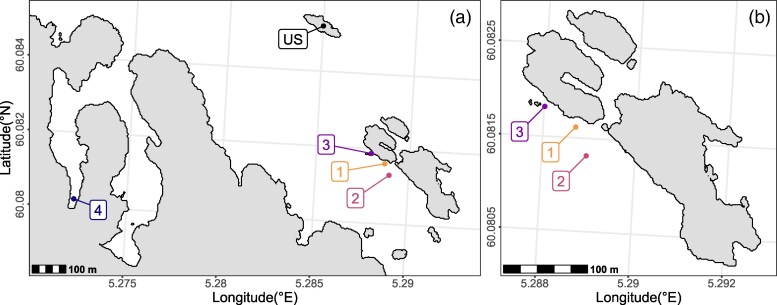
a) Locations of study sites, 1 to 3 = saltskjærholmane sites, 4 = kolbeinshamn bay sites, US, unfamiliar site. b) Higher resolution map of Saltskjærholmane showing sites 1 to 3). Elevation data courtesy of Høydedata (2024).

Data collection took place June 11 to 28, 2023, in the vicinity of Saltskjærholmane island (60°4′54.11″N, 5°17′24.219″E) and Kolbeinshamn bay (60°04′48.8″N, 5°16′20.3″E) in Austevoll, western Norway. The waters around Saltskjærholmane are protected from commercial wrasse fisheries while Kolbeinshamn is highly developed and generally unsuitable for commercial fishing practices. Between 1st May and 30th July, corkwing wrasse nest in high densities in both locations. In total, this study focused testing in 4 sites, 3 around Saltskjærholmane (1 to 3; Fig. 1) and 1 inside Kolbeinshamn bay (4; Fig. 1a). In addition, wrasse were caught and photographed in a 5th location (unfamiliar site—US; Fig. 1) as part of an ongoing mark-recapture study (Halvorsen et al. 2017, 2021). A total of 12 pairs of lateral photographs (4 photograph pairs for each morph) were fitted to the models for behavioral testing. The 3 main sampling locations are assumed to be different populations with little or no connectivity. From 2017 to 2019 mark-recapture surveys with similar sampling intensities were carried out around Saltskjærholmane and the US, of 2,850 recaptures only 15 individuals (0.005%) had crossed the 270 m of open waters separating the US and Saltskjærholmane (Halvorsen unpublished data). The high site fidelity and limited connectivity across open waters have also been demonstrated in other tagging studies conducted in nearby locations (Halvorsen et al. 2017, 2021).

#### Nest selection

The nests of corkwing wrasse form prominent structures (Potts 1985) that can be visually identified from the water surface via snorkeling surveys. All nests above a 5-m depth with an active nesting male were sampled at each site. Nests were marked using a weighted float marked with a unique numerical ID to identify corresponding nesting males.

#### Aggression tests

A pilot study conducted in 2022 demonstrated that an inanimate silhouette model would not elicit an agonistic response from nesting male corkwing wrasse unless affixed with a photograph of a conspecific (Ellis unpublished data). Following this a methodology was developed to assess the agonistic responses of nesting male corkwing wrasse to conspecific nest intruders (Ellis 2023).

**Figure 2:**
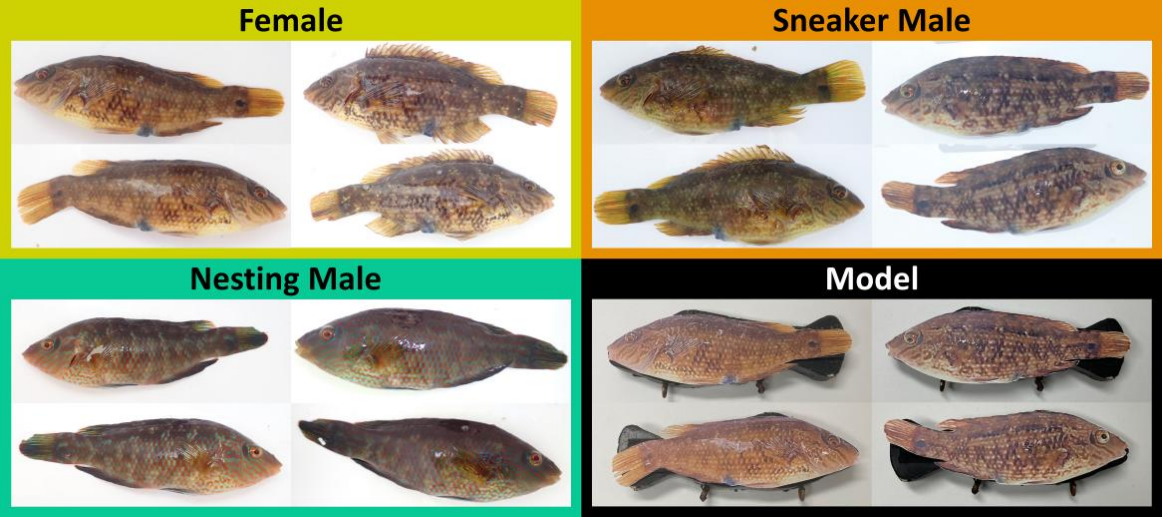
Images used as unfamiliar models depicting, 2 female (top left), 2 sneaker male (top right), and 2 nesting male (bottom left) corkwing wrasse, as well as the painted wooden model with the attached image of an unfamiliar female and unfamiliar sneaker male (bottom right).

Here we use an adaptation of this methodology to assess the agonistic responses of nesting males to unfamiliar female, rival nesting male and sneaker male nest intruders. An inanimate model consisting of a wooden silhouette (coated in Plasti Dip—Aerosol Spray, a waterproof, nontoxic, rubberized black paint) of a corkwing wrasse in a nonaggressive posture (lowered dorsal fin) was affixed with lateral photographs of each side of an unfamiliar individual of each morph. To control for any potential role of size dimorphism in morph recognition a model of total length 150 mm was used with photographs of individuals between 140 and 160 mm to maintain the correct body proportions. This size range is the optimum ecologically relevant size overlap between the morphs, encompassing both the natural size range of sneaker males and females while excluding smaller nonreproductively active nesting males (Figure S1). All photographs were resized to 150 mm and then printed on waterproof paper before being attached to the models prior to testing (Fig. 2).

Prior to aggression tests waterproof cameras (GoPro HERO9 and HERO10) attached to a weighted base were placed approximately 50 cm from a given nest entrance. Nesting males were given a minimum of 30 min prior to aggression tests to acclimate to the cameras and resume normal behavior. Recording commenced immediately prior to model presentation.

Prior to testing, model presentation order was randomized for each nest. Focal males were presented with each model for 5 min per test. Models were placed by a snorkeler in the nest entrance facing into the nest, resulting in the focal male leaving the nest area. To minimize the risk of habituation to the models, tests were performed a minimum of 1 h apart. A minimum of 2 sampling periods (wherein each focal male was shown a model of each morph once) were performed at each site (1 to 4) at a minimum of 1 week apart, with new photographs used for each sampling period to ensure unfamiliarity with no nesting male being presented with the same model twice. A total of 237 tests were performed across 63 nests. Time of aggression tests varied due to environmental conditions throughout the experiment, tests occurred from 9:00 to 20:00 with average time being 12:00, this variation was accounted for in the analyses.

### Data analyses

#### Data screening

Prior to video analysis recorded footage was screened for visibility. Recordings were excluded from further analysis if either the model was obscured from the camera, preventing recording of model response data, or the nesting males’ facial pattern was not visible at any point, making individual identification impossible. Unfortunately, due to poor visibility, session 1 at site 4 was excluded from final analysis, with an additional 11 videos excluded due to the model being obscured from the camera. Resulting in 178 videos across 47 nests for behavioral recording.

#### Nesting male identification

To control for nesting male movement between nests during the experiment and prevent pseudo-replication, all tested nesting males were identified through comparative image analysis of unique facial patterns using multiple images per individual and assigned a unique identification number. A total of 3 nesting males had been captured at multiple nests during recording, and 1 nest was used by 2 nesting males, all nesting males tested were included in final analysis resulting in 45 individual nesting males recorded over the 178 tests.

#### Behavioral recording

To quantify the responses of focal males to each morph, we used Behavioral Observation Research Interactive Software (Friard and Gamba 2016) for video analysis. Two measures of agonistic behavior were recorded: the interval between the male crossing the maximum horizontal extent of the nest post model placement and then attacking the model (attack latency), and the frequency of attacks within 90 seconds after the initial attack (attack frequency). An attack was defined as the focal male biting the inanimate model. Twenty-six nesting males showed no response to the model in at least 1 test for a total of 55 of 178 tests showing no response. To investigate this high proportion of no-response tests a binomial analysis was first performed on attack probability prior to the final analyses. However, as attack latency and attack frequency could not be calculated from no-response tests these observations were excluded resulting in a total of 123 observations across 38 individual nesting males for the final analyses.

#### Ethical note

All methods were approved by the University of Plymouth (Application ID: ETHICS-55 to 2023) and complied with Norwegian Food Safety Authority's animal welfare laws, guidelines and policies as approved by the Norwegian Animal Research Authority (Application ID: 8715, 15307, 29473 and 30760).

#### Statistical analysis

All statistical analyses were performed in R v.4.1.2 (R Core Team 2021). To investigate the relationship between *model type* (MT) and *attack probability* generalized linear mixed models (GLMMs) with a binomial distribution were fitted using the R package *glmmTMB* v.1.1.5 (Brooks et al. 2017). Following this additional GLMMs were fitted with a log-linked gamma distribution to investigate the relationship between *MT* and *attack latency*, and a quasi-poisson (negative binomial type 1) distribution to investigate the relationship with *attack frequency*.

Our global models (Equation 1) all included the fixed effects of *model type* (*MT*; female, sneaker male or nesting male), *test site* (*TS*; 1 to 4), *total model exposures* (*TE*; 1 to 17), and *test time* (*TT*; hour in day). *TS* was included as a fixed effect to account for any consistent differences in nesting male aggression due to spatial differences, while *total model exposures* was included to account for any difference in response to the model due to repeated exposures (eg habituation). Preliminary investigations suggest spawning activity peaks in the early morning, and early afternoon (Authors Personal Observation) therefore *TT* was included to account for any potential daily variation in aggression levels. An interaction effect was included between *MT* and *TS* to account for any spatial differences in agonistic responses to *MT*. *Focal nesting male identification number* (*MID*) and *test day* (*DiY*; day in year) were always included as random effects to account for known consistent individual differences in nesting male aggression (Ellis 2023) as well as any potentially shared environmental conditions that may influence nesting male aggression, (eg temperature, or turbidity).


R=MT×TS+TE+TT+(1|MID)+(1|DiY)

Equation 1: Global model structure. *R* = response variable, MT = model type, TS = test site, TE = Total model exposures, TT = test time, MID = focal nesting male identification number, DiY = day in year. Asterisks denote interactions.

Models were fitted with combinations of *model type*, *test site*, *test time*, and *total model exposures* according to our hypotheses (Burnham and Anderson 2002). Akaike information criterion corrected for small sample size (AICc) was used to identify the optimal models for each response variable (Burnham and Anderson 2002; Burnham et al. 2011; Symonds and Moussalli 2011), with a more complex model only favored over a simpler one if the AICc value of the more complex model was at least 3 points lower than the simpler version. Support for models lacking any combination of fixed and random effects would indicate no relationship with the response variables of interest.

Underlying statistical assumptions were assessed through graphical inspection, R packages *performance* v.0.13.0 (Lüdecke et al. 2021), *DHARMa* v.0.4.6 (Hartig 2022), and *plyr* v.1.8.8 (Wickham 2011) were used to generate plots of residuals against fitted values and covariates. The R package *performance* v.0.13.0 (Lüdecke et al. 2021) was also used to calculate intraclass correlation coefficients for random effects (Table S1). The model selection process is presented in full alongside coefficients of optimal models in the electronic supplementary material (Tables S1–S3).

## Results

**Figure 3:**
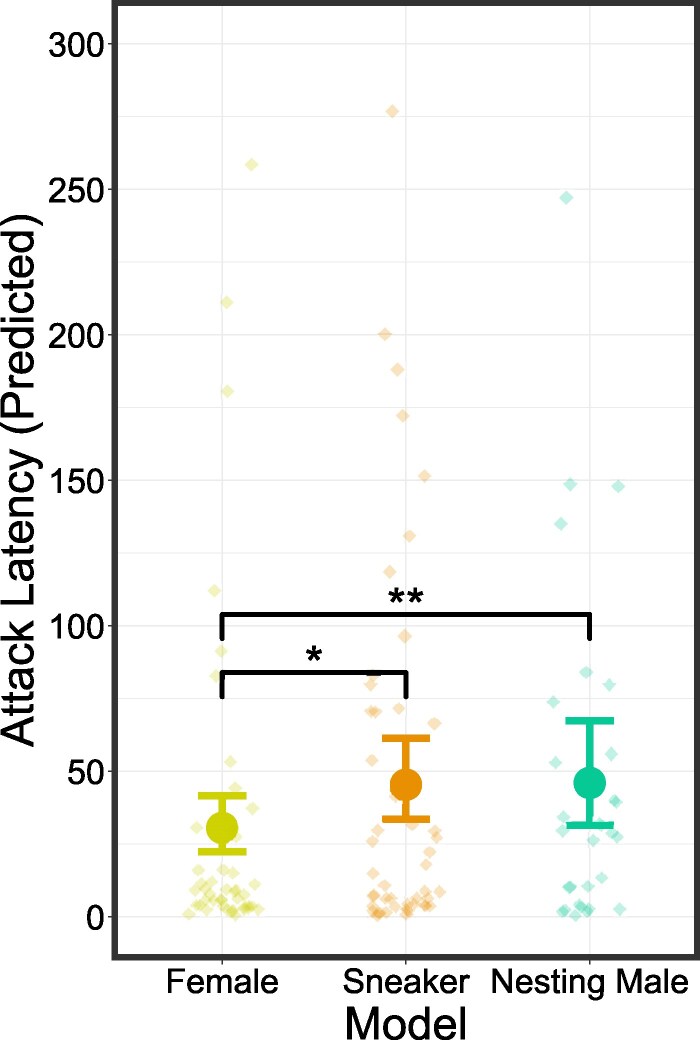
Model estimated relationships between *model type* and *attack latency*. Predicted values and corresponding 95% confidence intervals from optimal GLMMs are shown. Points show individual observations. Significant differences are shown in black (*n* = 123).

The optimal model for *attack probability* was the null model indicating no relationship with *model type*, *test site*, *total model exposures* or *test time*. The optimal model for *attack latency* contained a single fixed effect, *model type*, which influenced the attack latency of nesting males ($\chi_{2}^{2}$ = 8.6217, *P* = 0.013), with female models (F) provoking a lower attack latency compared with sneaker male (S) and nesting male (M) models (F—S: *P* = 0.038; F—M: *P* = 0.006; Fig. 3), with no difference between sneaker male and nesting male models (M—S: *P* = 0.278; Fig. 3). Neither *TS*, *total model exposures* or *TT* had an effect.

**Figure 4:**
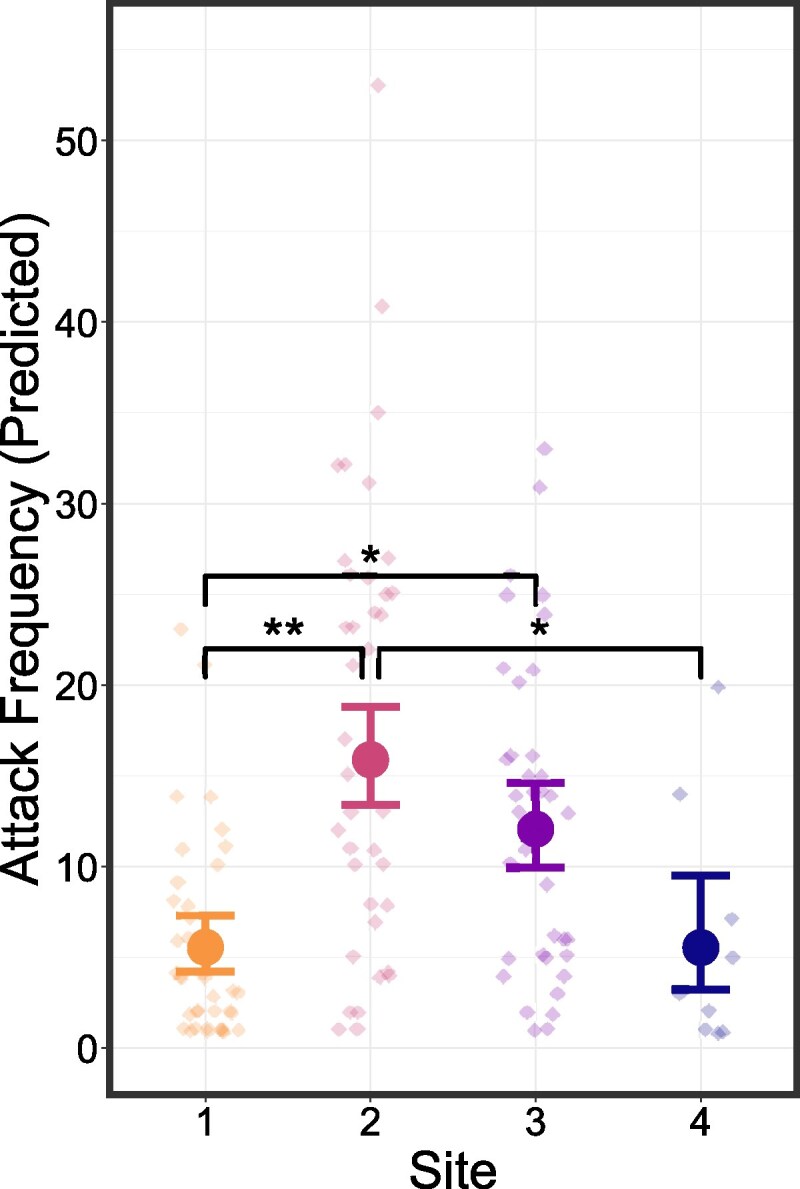
Model estimated relationships between *test site* and *attack frequency*. Predicted values and corresponding 95% confidence intervals from optimal GLMMs are shown. Points show individual observations. Significant differences are shown in black (*n* = 123).

Finally, test site was the only fixed effect included in the optimal model for *attack frequency* indicating no effect of *model type*, *total model exposures* or *test time*. *Test site* influenced attack frequency ($\chi_{3}^{2}$ = 11.302, *P* = 0.005) with nesting males at site one exhibited a lower attack frequency than at sites 2 and 3 (Site 1—Site 2: *P* = 0.002; Site 1—Site 3: *P* = 0.017; Fig. 4). At site 4 nesting males also exhibited a lower attack frequency than at site 2 (Site 4—Site 2: *P* = 0.047; Fig. 4), there were no other between site differences in response. Raw data distributions can be found in the supplementary material (Supplementary Figure S2).

## Discussion

The role of sensory modalities in recognizing cryptic morphs is an understudied aspect of ARTs. A potential consequence of this lack of information is that the selection of cues may not be suitable for answering key ecological questions. These include understanding species- and morph-specific social structures, the role of nonvisual mimicry in deception (Dalziell and Welbergen 2016) or how parentage impacts paternal investment (Neff and Sherman 2002). Using the corkwing wrasse as a model species, we investigated the importance of visual signals in facilitating sneaker recognition by nesting males. We predicted that if visual signals played no role in this recognition then the response of nesting males to sneaker and female models would be similar, and less agonistic than responses to a nesting male model due to both models being perceived as female. While if visual signals do play a role in recognition then we predicted that the sneaker and nesting male model would provoke a similar, more agonistic response than the female model. Surprisingly, we found that neither attack probability nor attack frequency was influenced by model type, with the latter being influenced by site, suggesting that social behavior and aggression levels can vary strongly at very small spatial scales. Regarding attack latency, female models surprisingly provoked the most agonistic response, being attacked significantly sooner than both nesting and sneaker male models, with no difference between the latency of attacks on the 2 male types. This result is in line with our second prediction and experimentally demonstrates the importance of the visual modality for sneaker recognition in nesting males.

In 31% of tests nesting males did not attack the model, surprisingly when investigated there was no support for an effect of test site, model type, total model exposure or test time on attack probability. Throughout the reproductive season nesting males undergo 2 to 3 nesting cycles during which they will construct a nest, court females and then care for the eggs until they hatch. These cycles are characterized by 2 to 3 days where spawning behavior is most frequent (Halvorsen in preparation). Prior to these peaks there are no eggs within the nest structure and minimal parental care behavior takes place. It is plausible that the nesting males that did not interact with the model were likely in this stage of the reproductive cycle, with the lack of eggs within the nest meaning the model posed no threat to the nesting males' fitness and therefore did not provoke an agonistic response.

Nesting males from 4 sites were sampled with nesting males at site 1 exhibiting significantly lower attack frequencies than nesting males at sites 2 and 3 (Fig. 4). This result is unexpected as sites 1, 2, and 3, should represent a homogenous population as they are not separated by the 30-m depth barrier characteristic of this species (Potts 1974). The nesting male population at Site 1 has been subject to passive behavioral monitoring since 2018 and was exposed to models of a nesting male in 2022 as part of a prior study (Ellis 2023). However, of the 14 nesting males used in the experiment at site 1, in the current study only 3 individuals were also present in 2022, suggesting that the observed low agonistic response is likely not due to habituation. This variation in response may instead be due to variations in unmeasured biotic and abiotic factors between sites. Sex ratios have profound impacts on a myriad of reproductive behaviors, such as courtship (de Jong et al. 2009; Clark and Grant 2010; Weir et al. 2011 Feb 1), parental care (Kokko and Jennions 2008; Fromhage and Jennions 2016; Jennions and Fromhage 2017), and sexual conflict (Grüter and Taborsky 2005; Weir et al. 2011; Chuard et al. 2016). The sex ratio of corkwing wrasse populations varies greatly at different spatial scales (Halvorsen et al. 2016, 2020), fishing intensities (Hanssen 2014) and depth (Halvorsen et al. 2020), therefore small scale differences in sex ratios between sites may account for part of the observed variation in response. In addition, reproductive behavior in other fish species is influenced by differences in factors such as substrate composition (Takemon and Nakanishi 1998; Galhardo et al. 2008, 2009; Júlio et al. 2021), nest density (Barnett and Pankhurst 1996; Mück et al. 2013), or food availability (Kolluru and Grether 2005) which can vary at fine spatial scales. Finally, anthropogenic disturbance may also play a role in the observed response; site 1 is exposed to increased human presence due to ongoing behavioral monitoring which has been shown to reduce fearfulness and antipredator responses (Frid and Dill 2002; Bessa and Gonçalves-de-Freitas 2014; Geffroy et al. 2015; Bessa et al. 2017), and increase stress (Baker et al. 2013; Bessa et al. 2017; Geffroy et al. 2018). While site 4, which also exhibited a significantly lower attack frequency than site 2, is subject to increased anthropogenic noise due to recreational boating activity which also increases stress (Popper and Hawkins 2019; Masud et al. 2020; Candolin and Rahman 2023) and alters reproductive behavior (Nedelec et al. 2017, 2022; Vieira et al. 2021). Thus, site differences in behavioral responses are not unexpected and are likely due to a complex interplay between many factors that vary across the sites present in this study, highlighting the importance of considering fine scale spatial variation when attempting to understand reproductive behaviors and systems exhibiting ARTs.

Surprisingly, no evidence was found to support the hypothesis that there are differences in attack frequencies between morphs. Potts (1974) observed intrasexual conflict in both nesting males and female corkwing wrasse, with territorial conflict between nesting males resulting in distinct visible injury and scarring but did not observe these interactions between males and females. Since this study, nesting males have been observed attacking both females and sneakers, resulting in similar injuries and scarring (Ellis personal obsservation). These conflicts are mostly brief, as the loser often rapidly retreats, with prolonged fights resulting in the observed damage to both individuals (Potts 1974). However, the models used in this study cannot retaliate and therefore pose minimal risk to the nesting male after the initial attack. The lack of an effect of model type is likely due to this, with the cost of frequent, rapid attacks being relatively low due to the inanimate nature of the model itself.

Nesting males exhibited longer attack latency when presented with the sneaker male model compared with the female model (Fig. 3). This result provides the first evidence that nesting males can differentiate between these cryptic morphs on visual information alone. Additionally, this also suggests that a lone female poses a higher risk to nesting male fitness than a lone sneaker, while this may appear counterintuitive it might be explained by natural behavioral differences between sneakers and females. Courtship in this species is often initiated by females approaching either the nesting male or nest from a lateral direction to signal her gravidity to the male (Potts 1974). After which, the male and female engage in rapid circular swimming, while vocalizing, during which gamete release occurs (Potts 1974; Bussmann et al. 2021). The models used in this study are inanimate and therefore cannot imitate these key aspects of courtship. During the spawning season, females are the only morph that have been observed to engage in egg predation, consuming the eggs within the nest when the nesting male is not present triggering a highly agonistic response (Karaszkiewicz 2020). The presence of an unresponsive female within the nest may therefore inadvertently imitate an egg predating female, prompting the observed low attack latency (rapid agonistic response).

Additionally, the attack latency of sneaker males was similar to the nesting male model, providing evidence that the risk of attacking a sneaker is comparable to attacking another nesting male, although as previously discussed it is unlikely that this is due to risk of injury from attacking a sneaker. In many fish species sneaker tactics are most effective during or immediately following the release of eggs by a female (Stoltz and Neff 2006b; Fitzpatrick 2020) due to a relatively low window of viability for eggs in most teleost fish species (Yamamoto 1962; Blaxter 1969, 2023). However, this fertilizability period can vary from minutes to hours depending on the species (Yamamoto 1962; Blaxter 1969, 2023). Assuming a short fertilizability period, the risk posed by the presence of a lone sneaker post-spawning (as simulated by the model) may be minimal compared with the energetic cost of a rapid attack, prompting the male to use his presence as a deterrent rather than immediately engaging in physical attacks. However, when startled by the nesting male, sneakers have been observed engaging in *streaking* behavior as they flee the nest, even long after eggs have been released, potentially fertilizing any unfertilized eggs reducing the fitness of the nesting male (Taborsky et al. 1987; Alonzo 2004; Stiver et al. 2018). If a longer fertilizability period is assumed then the observed attack latency may be due to the nesting male approaching the nest more cautiously to prevent this *streaking* behavior, again using his presence around the nest as a deterrent. Future research should be conducted to understand the fertilizability period in this species, in order to provide a key physiological context for the observed behavioral interaction.

Within these systems, traits that facilitate mimic detection are selected for in the territorial morph, while traits that facilitate detection avoidance are selected for in the alternative morph. As a result of this arms race, we would expect to find a high capacity for recognition in species exhibiting ARTs, especially in the territorial morph. However, this aspect of ART research is often neglected, with the cognitive abilities of the territorial morph, and the underlying sensory mechanisms underpinning them rarely being studied. Here we experimentally demonstrated that visual signals in isolation facilitate the recognition of unfamiliar sneakers by nesting male corkwing wrasse, providing the first evidence that visual information forms the mechanistic basis for recognition of cryptic morphs in a species exhibiting deception-based ARTs. These results suggest a fundamental misunderstanding of the mechanistic basis of sneaker mimicry in this system. Based on the high degree of female mimicry observed in sneaker males, we theorized that the successful deception of nesting males was dependent on the accurate imitation of the female visual morphotype, and that recognition by nesting males was facilitated through other modalities such as olfaction, behavioral cues or individual recognition of familiar sneakers. Here we provide evidence that nesting males exploit morphological differences between females and sneakers which have not yet been proven to be identifiable by human observers (Dipper and Pullin 1979; Uglem et al. 2000; Halvorsen et al. 2017). While the morphological features facilitating this recognition are currently unknown, preliminary results have shown that deep-learning based computer vision models can also differentiate sneakers and females with high precision. This can potentially enable identification of these key morphological characteristics using explainable AI or standard morphometric analyses (Sørdalen et al. in preparation). Additionally, these results suggest that the mimicry exhibited by sneakers is mechanistically less reliant on the visual modality for deception, and therefore more so on the other sensory modalities exhibited during courtship, such as display behaviors (Potts 1974) or acoustic signaling (Bussmann et al. 2021). Furthermore, while this study provides evidence that nesting males can recognize sneakers, there is considerable variation within the data. This variation suggests that recognition of sneakers may be situationally dependent or dependent on the individual, with some nesting males possessing a greater capacity for recognition. In order to better understand the ecological context of these findings further research should focus on the role that recognition plays in the courtship and reproduction of this species.

Using the corkwing wrasse as a model, this study reveals a fundamental knowledge gap in our understanding of the sensory modalities that maintain deception-based ARTs. These findings highlight the importance of using experimental approaches to quantifying the cognitive capabilities of teleost fishes, facilitating a greater understanding of their social behaviors, and the evolutionary processes that underpin them. Simultaneously our results demonstrate how the powerful selection pressures present in this system have produced a remarkable discriminative ability in this species, enabling not only the visual identification of a cryptic morph, but visual identification even in the absence of any behavioral or chemical cues.

## Supplementary Material

arag022_Supplementary_Data

## Data Availability

Analyses reported in this article can be reproduced using the data provided by (Ellis et al. 2026).
