# Supplementary material for: Visual cues elicit differential aggression towards female and female mimics in the corkwing wrasse
Source: Behav Ecol. 2026 Feb 25;37(3):arag022. doi: 10.1093/beheco/arag022 (PMC13017837; doi:10.1093/beheco/arag022)
Supplement: arag022_Supplementary_Data [file arag022_supplementary_data.pdf]

Supplementary Material

Intraclass correlation coefficients

Supplementary Table 1: Intraclass correlation coefficients (ICC) for random effects (MID = Male Identification Number, DiY = Day in Year), for each optimal model.

Response Variable	ICC MID	ICC DiY	ICC Adjusted	ICC Unadjusted
Attack Probability	0.490	0.233	0.722	0.722
Attack Latency	0.535	0.074	0.609	0.574
Attack Frequency	0.257	0.147	0.405	0.300

Candidate Model Sets

Supplementary Table 2: Candidate models to investigate the relationship between model type and attack probability. Optimal model used for inferences shown in bold italic. MT = Model Type, TS = Test Site, TE = Total Model Exposure, TT = Test Time, DiY = Day in Year, MID = Male Identification Number (n=178).

Model Structure	P	AICc	ΔAICc
**Attack Probability**			
1 + (1|DiY) + (1|MID)	***3***	***186.586***	***0.000***
ST + (1|DiY) + (1|MID)	6	188.667	2.081
MT + (1|DiY) + (1|MID)	5	190.607	4.021
TS + TE + (1|DiY) + (1|MID)	7	190.803	4.217
TS + TE + TT + (1|DiY) + (1|MID)	8	192.087	5.501
MT + TS + (1|DiY) + (1|MID)	8	192.675	6.090
MT + TE + (1|DiY) + (1|MID)	6	192.725	6.139
MT + TE + TT + (1|DiY) + (1|MID)	7	193.835	7.249
MT + TS + TE + (1|DiY) + (1|MID)	9	194.872	8.287
MT + TS + TE + TT + (1|DiY) + (1|MID)	10	196.094	9.509
MT x TS + TE + (1|DiY) + (1|MID)	15	201.507	14.921
MT x TS + TE + TT + (1|DiY) + (1|MID)	16	202.468	15.882

Supplementary Table 3: Candidate models to investigate the relationship between model type and attack latency. Optimal model used for inferences shown in bold italic. MT = Model Type, TS = Test Site, TE = Total Model Exposure, TT = Test Time, DiY = Day in Year, MID = Male Identification Number (n=123).

Model Structure	P	AICc	ΔAICc
**Attack Latency**			
**MT + (1|DiY) + (1|MID)**	**6**	**1114.236**	**0.000**
MT + TE + (1|DiY) + (1|MID)	7	1115.922	1.686
1 + (1|DiY) + (1|MID)	4	1118.097	3.861
MT + TE + TT + (1|DiY) + (1|MID)	8	1118.109	3.872
MT + TS + (1|DiY) + (1|MID)	9	1119.687	5.451
MT + TS + TE + (1|DiY) + (1|MID)	10	1121.531	7.295
TS + (1|DiY) + (1|MID)	7	1123.347	9.111
MT + TS + TE + TT + (1|DiY) + (1|MID)	11	1123.945	9.709
TS + TE + (1|DiY) + (1|MID)	8	1125.248	11.012
TS + TE + TT + (1|DiY) + (1|MID)	9	1127.223	12.986
MT x TS + TT + (1|DiY) + (1|MID)	16	1133.978	19.742
MT x TS + TE + TT + (1|DiY) + (1|MID)	17	1136.675	22.439

Supplementary Table 4: Candidate models to investigate the relationship between model type and attack frequency. Optimal model used for inferences shown in bold italic. MT = Model Type, TS = Test Site, TE = Total Model Exposure, TT = Test Time, DiY = Day in Year, MID = Male Identification Number (n=123).

Model Structure	P	AICc	ΔAICc
**Attack Frequency**			
**TS + (1|DiY) + (1|MID)**	**7**	**822.963**	**0.000**
TS + TE + (1|DiY) + (1|MID)	8	825.095	2.132
MT + TS + (1|DiY) + (1|MID)	9	825.949	2.986
TS + TE + TT + (1|DiY) + (1|MID)	9	827.294	4.331
MT + TS + TE + (1|DiY) + (1|MID)	10	828.151	5.188
1 + (1|DiY) + (1|MID)	4	829.008	6.045
MT + TS + TE + TT + (1|DiY) + (1|MID)	11	830.467	7.504
MT + (1|DiY) + (1|MID)	6	832.425	9.462
MT + TE + (1|DiY) + (1|MID)	7	834.219	11.256
MT + TT + TE + (1|DiY) + (1|MID)	8	836.508	13.545
MT x TS + TE + (1|DiY) + (1|MID)	16	840.439	17.476
MT x TS + TE +TT + (1|DiY) + (1|MID)	17	843.079	20.116

Raw Data

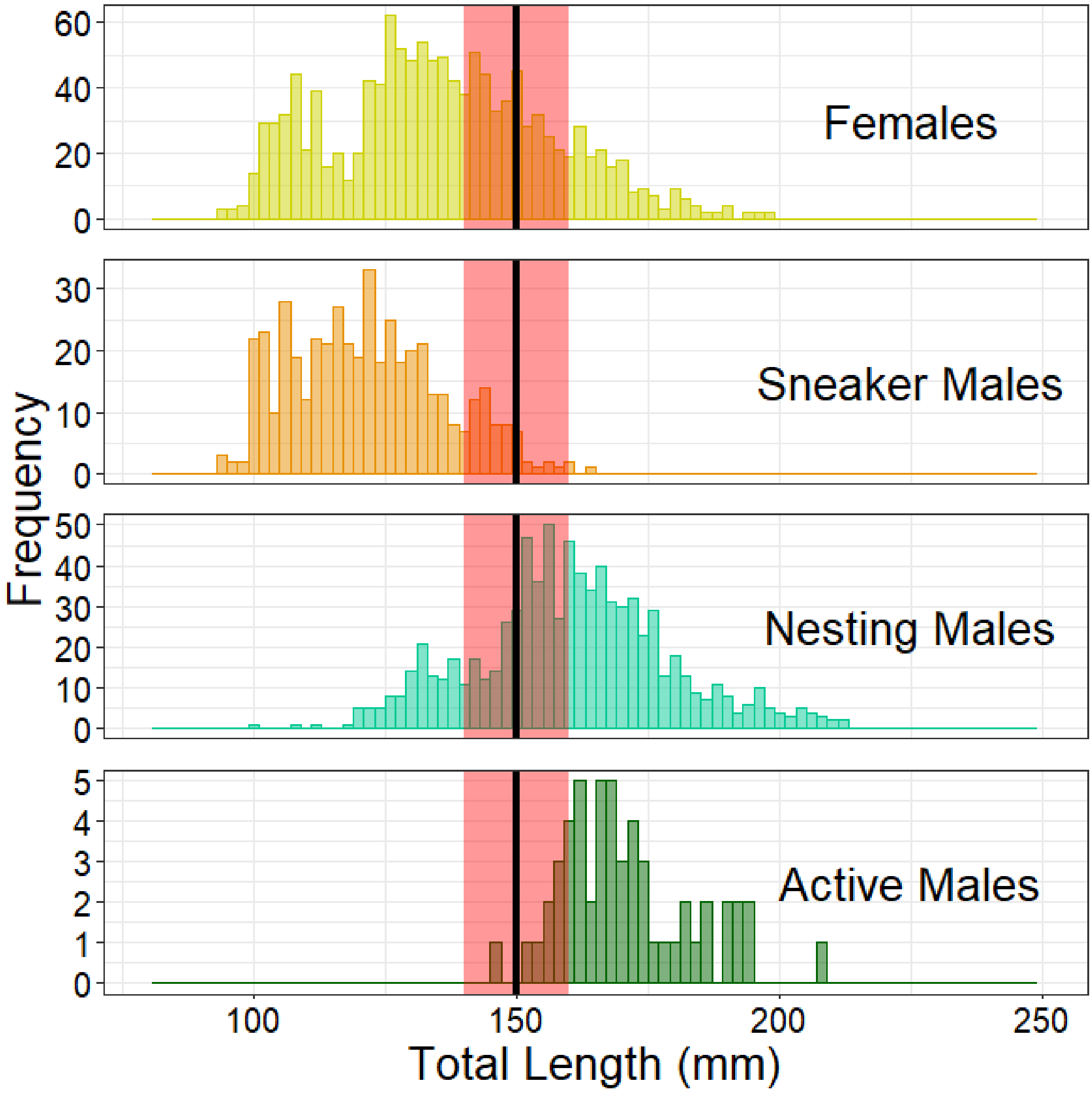

Supplementary Figure 1: Histograms depicting the frequency of spawning wrasses by total length for females (n=1260), sneaker males (n=465), all nesting males (n=811), and nesting males engaging in nesting behaviour (n=54). Red areas represent the size range of 140-160mm used to select the individuals whose photographs would be used for the inanimate model. The black line represents the size of the silhouette model to which photographs were attached.

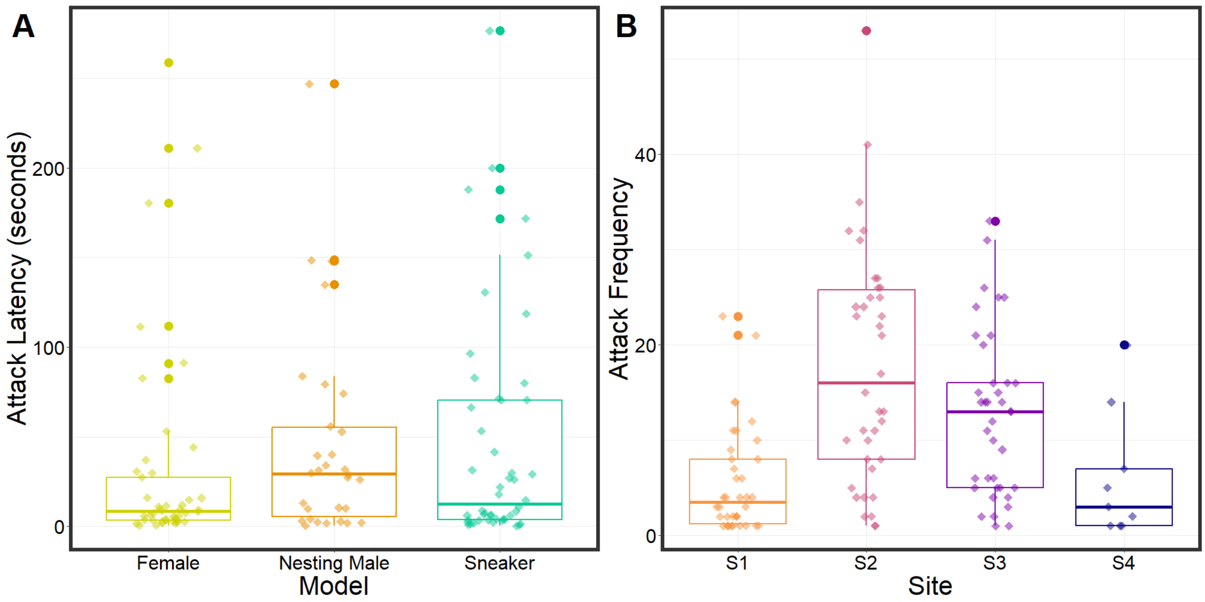

Supplementary Figure 2: Raw data for the relationships between: (A) Model type and attack latency. (B) Model type and attack frequency by site (n=123)
